# Superantigen-Producing *Staphylococcus aureus* Elicits Systemic Immune Activation in a Murine Wound Colonization Model

**DOI:** 10.3390/toxins7124886

**Published:** 2015-12-08

**Authors:** Choon K. Kim, Melissa J. Karau, Kerryl E. Greenwood-Quaintance, Ashenafi Y. Tilahun, Ashton Krogman, Chella S. David, Bobbi S. Pritt, Robin Patel, Govindarajan Rajagopalan

**Affiliations:** 1 Division of Clinical Microbiology, Department of Laboratory Medicine and Pathology, Mayo Clinic College of Medicine, 200 First Street SW, Rochester, MN 55905, USA; schenics77@gmail.com (C.K.K.); karau.melissa@mayo.edu (M.J.K.); piper.kerryl@mayo.edu (K.E.G.-Q.); pritt.bobbi@mayo.edu (B.S.P.); patel.robin@mayo.edu (R.P.); 2 Department of Immunology, Mayo Clinic College of Medicine, 200 First Street SW, Rochester, MN 55905, USA; aytilahun@mmm.com (A.Y.T.); Krogman.ashton@mayo.edu (A.K.); david.chella@mayo.edu (C.S.D.); 3 Division of Infectious Diseases, Department of Medicine, Mayo Clinic College of Medicine, 200 First Street SW, Rochester, MN 55905, USA

**Keywords:** HLA class II transgenic mice, *Staphylococcus aureus*, superantigen, skin infection, wound healing, inflammation

## Abstract

*Staphylococcus aureus*, the most common cause of wound infection, produces several exotoxins, including superantigens (SAgs). SAgs are the potent activators of the immune system. Given this unique property, we hypothesized that SAgs produced by *S. aureus* in wounds would have local, as well as systemic immunologic effects. We tested our hypothesis using a novel staphylococcal skin wound infection model in transgenic mice expressing HLA-DR3. Skin wounds were left uninfected or colonized with *S. aureus* strains producing SAgs or an isogenic strain not producing any SAg. Animals with wounds challenged with SAg-producing *S. aureus* had increased morbidity and lower serum IL-17 levels compared to those challenged with the SAg non-producing *S. aureus* (*p* = 0.027 and *p* = 0.032, respectively). At Day 8 following microbial challenge, compared to mice with uninfected wounds, the proportion of Vβ8^+^CD4^+^ T cells was increased, while the proportion of Vβ8^+^CD8^+^ T cells was decreased only in the spleens of mice challenged with SAg-producing *S. aureus* (*p* < 0.001). No such changes were measured in mice challenged with SAg non-producing *S. aureus*. Lungs, livers and kidneys from mice challenged with SAg-producing, but not SAg non-producing, *S. aureus* showed inflammatory changes. Overall, SAg-mediated systemic immune activation in wounds harboring *S. aureus* may have clinical implications.

## 1. Introduction

*Staphylococcus aureus* is a normal resident flora of the skin of even healthy individuals [1]. However, it is also the most common cause of wound infections [2]. *S. aureus*-infected wounds are characterized by purulence, which is a result of neutrophil recruitment to the wound site [3]. Not surprisingly, *S. aureus* has evolved strategies to subvert attack by the neutrophils and other innate cells that form the first line of defense. Examples include the extracellular adhesion protein (Eap) [4], chemotaxis inhibitory protein of *S. aureus* (CHIPS) [5] and converting enzymes to evade neutrophil extracellular traps (NET) [6]. In addition to these above-mentioned factors, *S. aureus* produces several enzymes and exotoxins, which may also contribute to staphylococcal immune evasion and the immunopathogenesis of staphylococcal wound infection [7]. The superantigen exotoxins of *S. aureus* may be important for these, because of their unique biological functions.

Staphylococcal superantigens (SAgs) are a family of polypeptide exotoxins produced by *S. aureus.* SAgs are known for their ability to nonspecifically activate a large percentage of T cells by binding to the MHC II molecules on antigen-presenting cells and to certain variable regions of the T cell receptor β chains (Vβ) (or TCR Vα for Staphylococcal enterotoxin H) [8,9]. Given that a large percentage of *S. aureus* isolated from skin and soft tissue infections can produce SAgs [10,11] and the known immunostimulatory properties of SAgs, we hypothesized that SAgs may not only modulate the local immune response in wounds harboring *S. aureus*, but may also exert some systemic immune effects due to absorption of SAg from the disrupted epithelial barrier in the wounded skin.

It is a challenge to study the local, as well as the systemic effects of SAgs produced by *S. aureus* in wounds due to a lack of suitable small animal models. While conventional mouse models have been used to study wound infections caused by *S. aureus*, conventional mice do not respond robustly to SAgs. This is due to poor binding of SAgs to murine MHC class II molecules [12,13]. On the other hand, SAg binds more avidly to human MHC class II molecules. Therefore, we have developed a strategy to overcome this limitation by transgenically expressing human MHC class II (HLA-DR3) molecules in mice [14]. Given that the binding sites on TCR Vβ are either conserved or the amino acids are structurally similar between mice and humans [15], HLA-DR3 transgenic mice respond strongly to many SAgs, including staphylococcal enterotoxin B (SEB), even though the T cells are of mouse origin [12]. Herein, we describe a novel murine skin wound infection model in humanized HLA-DR3 transgenic mice to investigate the local and systemic consequences of wound infection by SAg-producing *S. aureus*.

## 2. Results

### 2.1. SAg-Producing S. aureus Delays Wound Healing

**Figure 1:**
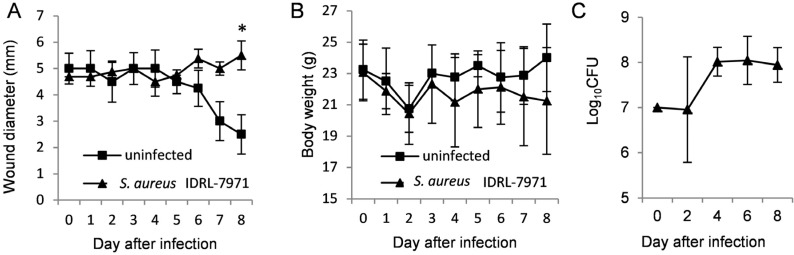
Impact of *S. aureus* colonization on wound healing: comparison of wound sizes (**A**) and body weights (**B**) between uninfected DR3 mice (■, *n* = 4) and DR3 mice challenged with IDRL-7971 (▲, *n* = 16). Wound size was larger in the DR3 mice challenged with IDRL-7971 than the uninfected mice. After the initial 10^7^ CFU were inoculated on the wound, the mean bacterial quantities of skin wounds were measured from three sacrificed mice at Days 2, 4, 6 and 8, respectively (**C**). The mean and SD are depicted. *p*-values were calculated by the Mann-Whitney U-test; * *p* < 0.05 at each time point.

Experimental skin wounds were monitored every day. The differences in mean wound sizes between DR3 mice challenged with *S. aureus* IDRL-7971, which produces the SAgs SEA and SEB (*n* = 16), and uninfected controls (*n* = 4) are shown in Figure 1A. The mean wound size of DR3 mice challenged with *S. aureus* IDRL-7971 was larger than that of the uninfected controls at Day 8 (*p* = 0.002). The body weight of mice with infected wounds was lower and failed to reach their pre-infection weights by Day 8, while mice with uninfected wounds maintained their body weight or even gained weight by the time the wounds were healed (Figure 1B). The mean bacterial quantity in the skin wound increased by one order of magnitude on Days 2 to 4 in DR3 mice challenged with *S. aureus* IDRL-7971, with no change in the bacterial quantity from Day 4 to 8 (Figure 1C). No blood or organ homogenate cultures collected at the time of sacrifice grew *S. aureus* in DR3 mice with *S. aureus* IDRL-7971-colonized wounds, providing evidence that the infection remained localized to the wound.

### 2.2. Wound Healing Was Similar in Wounds Challenged with SEB-Producing or SEB Non-Producing S. aureus

**Figure 2:**
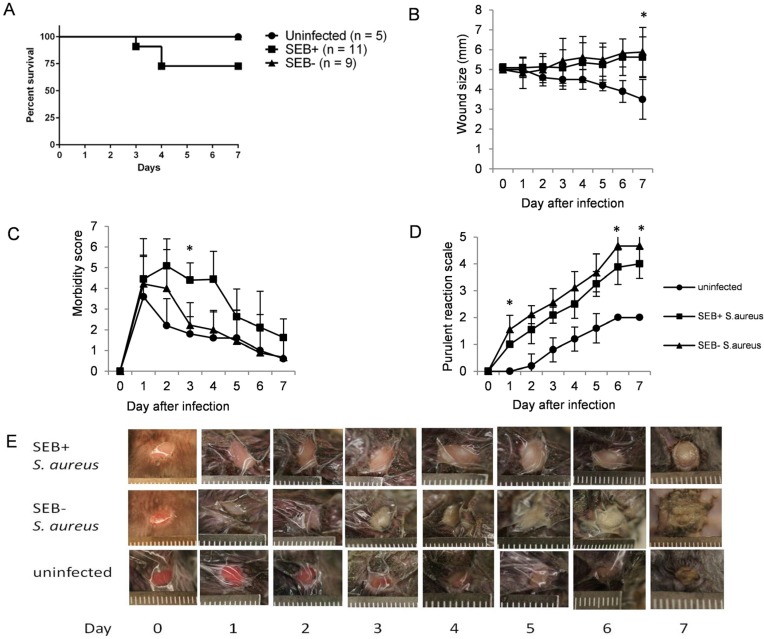
Impact of superantigen (SAg) produced by *S. aureus* on wound healing: mortality (**A**), comparison of wound size (**B**), the morbidity score (**C**) and the exudate scales (**D**) among the mice challenged with SEB-producing *S. aureus* RN6734/pRN7114 (■, *n* = 11) or *S. aureus* not producing SEB, RN6734/pRN7116 (▲, *n* = 9) and the uninfected mice (●, *n* = 5). (**E**) The wound of the mice with RN6734/pRN7114 (upper row) and RN6734/pRN7116 (middle row) and the uninfected mice (lower row). The mean and SD are depicted. *p*-values were calculated by the Mann-Whiney U-test; * *p* < 0.05 at each time point.

To further differentiate the effects of infection *per se* from the effects of SAg on wound healing, we challenged wounds with isogenic strains of *S. aureus*, one capable of producing SAg and the other not. The mean wound size of DR3 mice challenged with *S. aureus* RN6734/pRN7114 (SEB+) or RN6734/pRN7116 (SEB−) and uninfected controls is shown in Figure 2B. There was no difference in the wound size in DR3 mice challenged with the two *S. aureus* strains. DR3 mice challenged with either SEB+ *S. aureus* RN6734/pRN7114 (*n* = 8) or SEB− *S. aureus* RN6734/pRN7116 (*n* = 9) had delayed wound healing compared to uninfected controls (*n* = 5) (*p* = 0.005 and *p* = 0.003, respectively) on Day 7. This suggests that infection with *S. aureus* *per se* delays wound healing, but that SAgs do not directly affect this process, at least wound size.

### 2.3. Mice Challenged with SEB-Producing S. aureus Were Sicker and Had Delayed Wound Exudate Formation Compared to Those Challenged with SEB Non-Producing S. aureus

Among the 11 DR3 mice challenged with SEB+ *S. aureus* RN6734/pRN7114, eight survived until Day 7. Two were dead on Days 3 and 4, respectively, and one was euthanized on Day 4 due to a morbidity score of 10 (Figure 2A). All nine DR3 mice challenged with SEB− *S. aureus* RN6734/pRN7116 survived until Day 7. DR3 mice challenged with RN6734/pRN7114 (SEB+) had higher morbidity scores than those challenged with RN6734/pRN7116 (SEB−) on Day 3 (*p* = 0.043; Figure 2C). Surprisingly, on Days 1, 6 and 7, the exudate score was lower in DR3 mice challenged with SEB+ *S. aureus* RN6734/pRN7114 compared to those challenged with SEB− *S. aureus* RN6734/pRN7116 (*p* = 0.038, 0.036 and 0.046, respectively; Figure 2D,E).

By linear regression analysis, the increase in the exudate score of the DR3 mice challenged with SEB+ *S. aureus* RN6734/pRN7114 (SEB+) was delayed compared to those challenged with SEB− *S. aureus* RN6734/pRN7116 (*p* < 0.001). There was a tendency for DR3 mice with high morbidity scores to have less wound exudate than with low morbidity scores. Nonetheless, there was no difference in the mean bacterial quantities in the skin wounds between the mice challenged with SEB+ *S.* *aureus* RN6734/pRN7116 and SEB− *S. aureus* RN6734/pRN7114 (3.03 × 10^8^ CFU *vs*. 1.72 × 10^8^ CFU, respectively). No blood and organ homogenate cultures collected at sacrifice grew *S. aureus* in DR3 mice challenged with either SEB+ or SEB− *S. aureus*. In an additional experiment using an inoculum of 10^8^ CFU, there was no difference in mortality between DR3 mice challenged with 10^7^ and 10^8^ CFU of *S. aureus* RN6734/pRN7116 (3/11 *vs.* 1/7, respectively).

### 2.4. Serum IL-17 Was Lower in the Mice Challenged with the SEB-Producing Compared to the Non-SEB-Producing S. aureus

**Figure 3:**
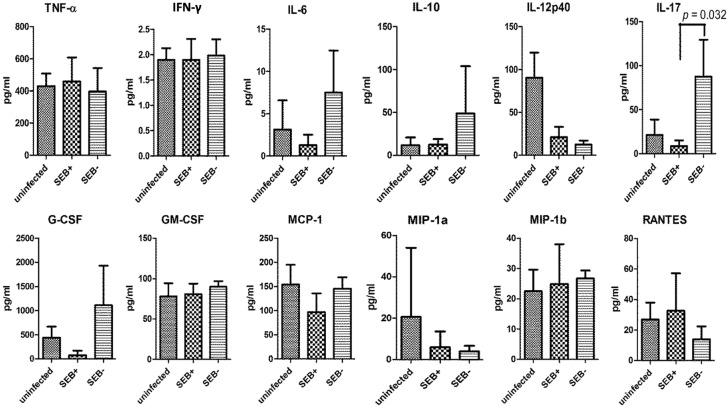
Impact of SAg produced by *S. aureus* colonizing wounds on systemic cytokine/chemokine levels: levels of serum cytokines at the time of sacrifice among DR3 mice challenged with RN6734/pRN7114 (*n* = 3), RN6734/pRN7116 (*n* = 3) and uninfected DR3 mice (*n* = 3). The level of IL-17 was significantly decreased in mice challenged with RN6734/pRN7114 (SEB-producing) than RN6734/pRN7116 (SEB non-producing). *p*-values were calculated by the Student *t*-test.

Since SEB+ *S. aureus* RN6734/pRN7116 inhibited the exudative reaction, which is presumably mediated by neutrophils, and since IL-17 is a crucial cytokine that has been shown to regulate cutaneous responses to *S. aureus* [16], we next investigated the serum levels of IL-17 and other cytokines among DR3 mice challenged with SEB+ *S. aureus* RN6734/pRN7116 (*n* = 3) or SEB− *S. aureus* RN6734/pRN7114 (*n* = 3) and uninfected controls (*n* = 3) using sera collected at the time of sacrifice (Figure 3). The levels of IL-2, IL-3, IL-4 and IL-5 were below the limit of detection in all three groups. The levels of TNF-α, IFN-γ, IL-6, IL-10, IL-12p40, MCP-1, MIP-1a, MIP-1b, GM-CSF and RANTES were similar between the DR3 mice challenged with SEB+ *S. aureus* RN6734/pRN7116 and those with SEB− *S. aureus* RN6734/pRN7114. Interestingly, the IL-17 level was lower in DR3 mice challenged with SEB+ *S. aureus* RN6734/pRN7114 than those with SEB− *S. aureus* RN6734/pRN7116 (*p* = 0.032). Moreover, IL-6, a cytokine associated with the differentiation of Th0 cells to Th17 cells producing IL-17, was slightly higher in mice challenged with *S. aureus* SEB− RN6734/pRN7116, although this did not attain statistical significance. Furthermore, G-CSF, a cytokine responsible for neutrophil production and maturation, was elevated slightly, but not significantly, in mice challenged with SEB− *S. aureus* RN6734/pRN7116, which had elevated IL-17 and greater apparent neutrophil recruitment to the wound (*i.e.*, purulence).

### 2.5. SAg-Producing S. aureus in Skin Wounds Causes Expansion of Splenic CD4^+^ T Cell and Multi-Organ Inflammation

**Figure 4:**
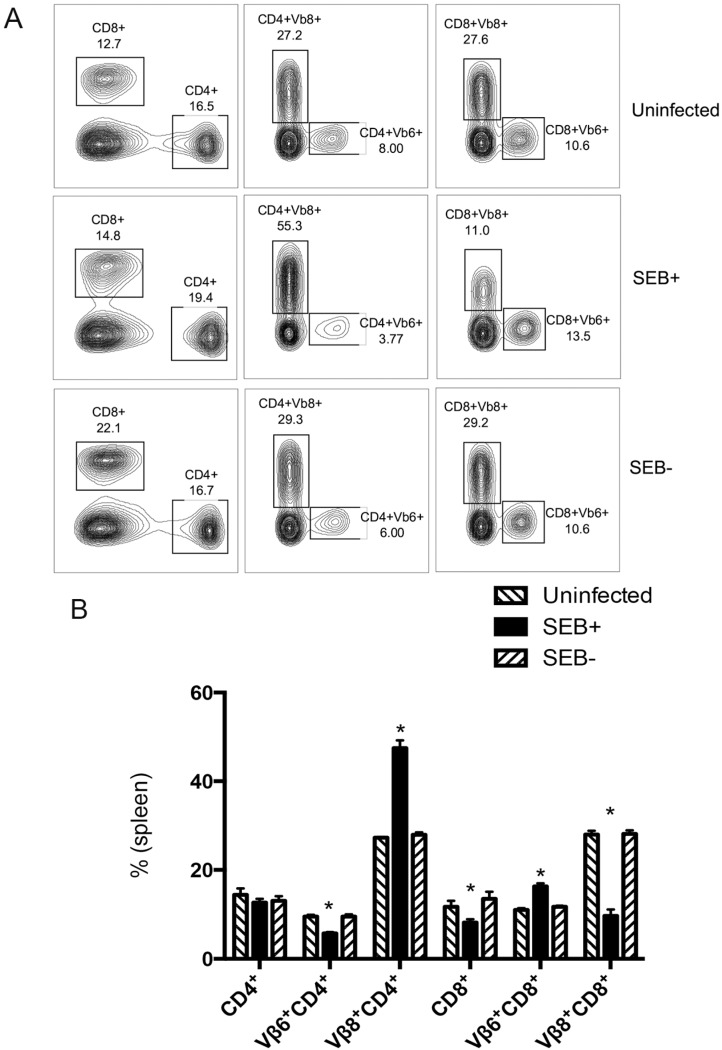
Systemic effects of SAg produced by *S. aureus* colonizing wounds on splenic T cell numbers: the distribution of T cells in spleens of DR3 mice challenged with SEB+ RN6734/pRN7114 (SEB producing), SEB− RN6734/pRN7116 (SEB− non-producing) or uninfected was analyzed by flow cytometry using FITC-conjugated anti-Vβ6 or anti-Vβ8, phycoerythrin-conjugated CD4 and PerCP-conjugated CD8 antibodies. The proportion of CD4^+^ and CD8^+^ T cells expressing TCR Vβ8 and TCR Vβ6 was significantly altered in mice infected with SEB producing *S. aureus* compared to mice infected with *S. aureus* not capable of producing SEB, which mimicked the uninfected mice. (**A**) Representative dot plots; (**B**) the means and standard errors are depicted. * *p*-value less than 0.001; *p*-values were calculated by the Student *t*-test.

Since the skin barrier is effectively disrupted in mice with skin wounds, we hypothesized that small amounts of SAg produced in the wounds may be absorbed through the skin. Given the property of SAg to activate T cells, antigen-presenting cells and other cells of the immune system either directly or through cytokines/chemokines, we theorized that this might be sufficient to activate the immune system and exert some immunopathology, even though the organism is localized to the skin wound and not demonstrable in blood or other tissues. To investigate systemic immune activation, the distribution of CD4^+^ and CD8^+^ T cells expressing TCR Vβ6 or Vβ8 in the spleens was analyzed by flow cytometry. It should be noted that SEB binds to T cells expressing TCR Vβ8 [15]. Therefore, changes in the percentages of T cells expressing TCR Vβ8 and Vβ6 in spleens would be indicative of the involvement of SAg (*i.e.*, SEB in our model). In our experiment, as is evident from Figure 4, the proportion of CD4^+^ and CD8^+^ T cells expressing TCR Vβ8 and Vβ6 was significantly different in the spleens of mice challenged with *S. aureus* SEB+ RN6734/pRN7114 compared to DR3 mice with wounds colonized with SEB− *S. aureus* RN6734/pRN7116, which was comparable to mice with uninfected wounds (*p* < 0.001, *p* < 0.001; Figure 4) [17,18]. This observation combined with our finding that we were not able to culture any bacteria in blood, spleen or any tissue indicated that SEB produced in the skin was able to cause systemic immune activation.

**Figure 5:**
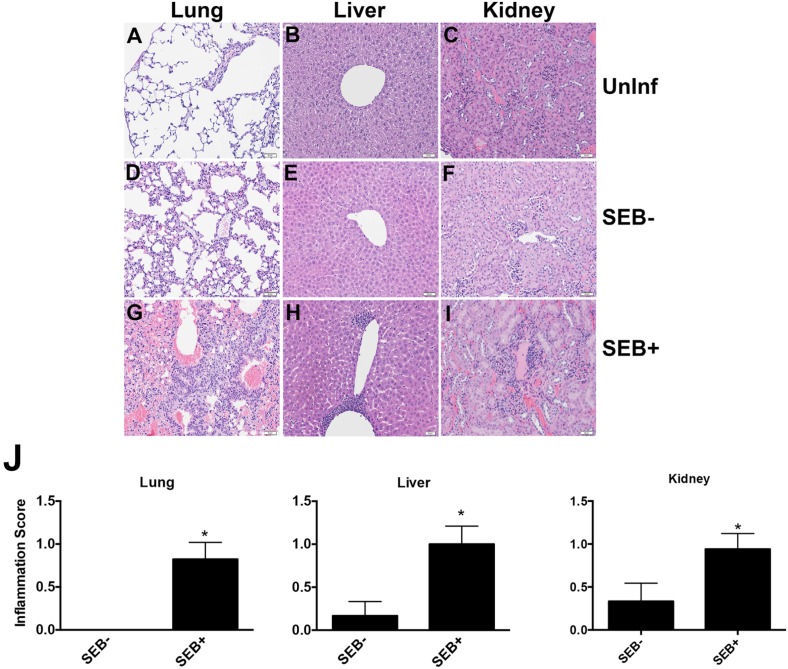
Systemic effects of SAg produced by *S. aureus* colonizing wounds: lungs, livers and kidneys from DR3 mice with wounds that were colonized with either RN6734/pRN7114 or RN6734/pRN7116 were stained with H&E. The extent of inflammation was scored in a blinded fashion by comparing with tissue sections obtained from DR3 mice with uninfected wounds. (Panels **A** through **I**) Representative sections. Scale: White bars in histology sections correspond to 50 μM. Panel **J** depicts inflammation scores. For each group, six to 16 individual sections from different mice were examined. The inflammation score was higher in organs from mice colonized with RN6734/pRN7114 (SEB-producing *S. aureus*) than RN6734/pRN7116 (SEB non-producing *S. aureus*). The means and standard deviation are depicted. *p*-values were calculated by the Student *t*-test, * *p* < 0.05.

Histopathological evaluation of tissues from different mice groups showed the presence of inflammatory cell infiltrates in the livers, kidneys and lungs only in mice challenged with SEB+ *S. aureus* RN6734/pRN7114 compared to uninfected controls. Tissues from mice challenged with SEB− *S. aureus* RN6734/pRN7116 showed minimal or no inflammation (Figure 5) [17,18]. The presence of these inflammatory changes only in mice challenged with SEB-producing *S. aureus* RN6734/pRN7114, but not its isogenic RN6734/pRN7116 strain not producing SEB, strongly suggests the involvement of SEB in eliciting this systemic inflammatory process. These systemic findings were also seen in DR3 mice challenged with the clinical *S. aureus* isolate IDRL-7971.

## 3. Discussion

The most common cause of wound infection/colonization is *S. aureus*, a member of the commensal flora in the superficial skin [19,20]. *S. aureus* uses several secreted and cell wall-bound factors to evade or subvert the immune system, and SAgs are one among them [21,22]. SAgs are known for their ability to robustly activate the immune system, and their contribution to the immunopathogenesis of serious life-threatening infections is well known. Herein, we demonstrate for the first time that SAgs produced by *S. aureus* localized to skin wounds may modulate the immune response, both locally, as well as systemically. Locally, at the macroscopic level, we demonstrated apparent decreased purulence in wounds of DR3 mice challenged with SEB-producing compared to an isogenic SEB non-producing *S. aureus*. In support of our findings, Vojtov *et al.* have reported that a SAg-positive *S. aureus* strain caused less skin inflammation than a SAg-negative *S. aureus* in a murine skin abscess model [23].

There could be several possible immunologic explanations by which SAg could modulate local inflammatory response in addition to suppression of the synthesis of other exotoxins, such as lipase, which is chemotactic for neutrophils [23]. First, SEB predominantly induces Th1 polarization of naive CD4^+^ T cells, and Th1-related cytokines, such as IFN-γ, inhibit Th17 differentiation [24,25]. We have also shown that HLA-DR3 mice produce much higher levels of IFN-γ than IL-17 early on following stimulation with purified SEB or following infection with *S. aureus* producing SEB [12,14,18,26]. Therefore, SEB could suppress IL-17 through IFN-γ, resulting in reduced local inflammatory reaction in wounds colonized with *S. aureus* producing SEB. In this study, the serum level of IL-17 was also decreased on Day 7 in the DR3 mice challenged with SEB-producing *S. aureus* compared to non-SEB-producing *S. aureus*. This suggests that while *S. aureus* can induce the production of IL-17 by γδ T cells [16], the presence of SAgs may inhibit the production of IL-17 through induction of Th1 cytokines. While not investigated in this study, SAg might inhibit IL-17 production through other cytokines, such as IL-19, IL-20 and IL-24 [27]. Second, CD4^+^ T cells activated by SAgs may suppress innate immune responses, such as chemokine secretion by macrophages [28,29]. Third, T regulatory cells induced by SAgs may directly suppress activation of innate immune cells [30,31]. Overall, we hypothesize that SAgs may play a role in inhibition of neutrophil recruitment based on one or more of the above mechanisms. The ability to produce SAgs may confer to *S. aureus* the ability to evade innate immunity, rendering it a successful pathogen in wound infections. However, we have evaluated IL-17 levels only on Day 8, when the exudative reaction was highest. Additional experiments may be needed to determine the temporal kinetics of cytokine/chemokine induction by SAg produced by *S. aureus* infecting the wounds at the molecular level.

While skin wound infection with *S. aureus* is common, the systemic effects of SAg produced by *S. aureus* localized to wounds have not been reported. Our study, to our knowledge, for the first time showed that SAg could be absorbed through damaged skin and cause systemic immune activation. We also believe that *S. aureus* colonizing the wounds is producing only small amounts of SAg. As a result, it is not able to induce a massive systemic cytokine storm that is classically seen in patients with sepsis or septic shock, which is also associated with high mortality. As SAg causes TCR Vβ-dependent, but cognate antigen-independent T cell activation, chronic exposure to small amounts of SAg causes continued expansion of CD4^+^ T cells expressing TCR Vβ8 that are reactive to SEB. Therefore, the percentage of CD4^+^ T cells expressing TCR Vβ8 goes up. As it is a measure of percentage, the percentage of CD4^+^ T cells bearing TCR Vβ6 that do not bind to SEB goes down correspondingly. Chronic antigenic stimulation has been shown to induce exhaustion and deletion of mainly the CD8^+^ T cells in several tumor models and persistent viral infections [32,33,34]. As SAg can also activate CD8^+^ T cells, we believe that chronic stimulation by SAg leads to deletion of CD8^+^ T cells expressing TCR Vβ8 that reacts with SEB. Therefore, the percentage of CD8^+^ T cells expressing TCR Vβ8 that binds to SEB goes down, while the percentage of CD8^+^ T cells expressing TCR Vβ6 that does not bind to SEB goes up correspondingly. Chronically-activated CD4^+^ T cells and other immune cells migrate through various organs and tissues, looking for their cognate antigen or the inciting stimulus, resulting in infiltration of various tissues/organs with inflammatory cells. Given the chronicity of wounds in certain patients, e.g., diabetics, recurrent exposure to SAg through skin wounds might exert various systemic immune effects and should be explored. Our HLA class II transgenic mice would be ideal for such investigations. In conclusion, staphylococcal SAgs may play a role in wound healing by acting locally, as well as systemically.

## 4. Materials and Methods

### 4.1. S. aureus Stra12ins

Three *S. aureus* strains were studied. IDRL-7971, isolated from a nasal swab, produces only the SAgs staphylococcal enterotoxins A and B (SEA and SEB), as shown by PCR and confirmed by ELISA [18,35,36]. RN6734, containing the intact cloned *seb*, pRN5543::*seb* (pRN7114), produces only SEB (SEB+). We also used its derivative with a large 3ʹ deletion, pRN5543::*seb*(b.2) (pRN7116), which does not produce any SAg, including SEB (SEB−). The latter two strains were generous gifts from Richard Novick, New York University Medical Center, New York, NY, USA. The RN6734 derivatives were grown in trypticase soy broth supplemented with chloramphenicol (20 µg/mL) [23,35,36]. The Institutional Animal Care and Use Committee approved all animal experiments, and as the experiments involve SAg, our work was also approved by the Biosafety Committee.

### 4.2. Mice

HLA-DR3 transgenic mice (DR3 mice), expressing functional HLA-DRA1*0101 and HLA-DRB1*0301 transgenes on the complete murine MHC class II-deficient background (AE°), were studied [37]. We have previously demonstrated that HLA-DR3 transgenic mice respond robustly to purified SAgs, as well as to SAg produced by live bacteria following infection/colonization [12,14,17,18,36]. Experimental protocols were approved by the Mayo Clinic Institutional Animal Care and Use Committee. Protocol approval number, A37613.

### 4.3. Wound Infection Model

Mice were anesthetized by intraperitoneal injection of ketamine (90 mg/kg) and xylazine (10 mg/kg). The skin of the dorsum of the mice was shaved and disinfected with 2% chlorhexidine and an alcohol swab. Subsequently, a sterile 5-mm diameter circular full thickness skin wound was created using a skin puncture biopsy tool (Acuderm Inc., Fort Lauderdale, FL, USA). Excised skin was homogenized and quantitatively cultured. Using a Pipetman micropipette (Gilson, Inc., Middleton, WI, USA), 10^7^ CFU *S. aureus* in 10 µL of normal saline were inoculated onto the wound bed. Vehicle alone was inoculated in the uninfected control mice. To prevent secondary bacterial contamination and allow visualization of the wound bed, semi-occlusive transparent Tegaderm^®^ (3M, St. Paul, MN, USA) was placed over the wound using liquid adhesive Mastisol^®^ (Eloquest Health care, Ferndale, MI, USA) [38]. Photographs of the wound were taken, and the diameter of the wound was recorded daily. On a daily basis, a scoring scale assessing changes in body weight, movement when touched, eye opening and body curling when grabbed by the end of the tail and lifted, were utilized to record morbidity (Table 1). Mice with morbidity scores of 10 were euthanized. Furthermore, on a daily basis, the amount of exudate in the wound was graded by observing the wound bed through the Tegaderm^®^, using the scoring scale in Table 1. At the time of sacrifice, small pieces of lung, liver, kidney and skin were collected and prepared for histopathology. Spleens were collected in phosphate-buffered saline (PBS) and prepared for flow cytometric analysis. The skin surrounding the wound was excised using a 10-mm skin puncture biopsy and quantitatively cultured. Livers, kidneys and lungs were homogenized and quantitatively cultured. Blood was collected by cardiac puncture and cultured for 5 days. Serum was separated from blood and stored at −80 °C for cytokine assay.

**Table 1:** Scoring system and scale.

**Mouse Morbidity Score**				
**Feature**	**0**	**1**	**2**	**3**
Body weight loss	No change	0%–9%	10%–19%	≥20%
Eye opening	Fully open with no crust	Incomplete but more than half open	Less than half open with crust	Closed
Movement at touch	Running and jumping	Running but not jumping	Walking	No movement with hunched posture
Curling	Active curling (≥2 per second)	Decreased or absent curling (0–1 per second)	Not Applicable	Not Applicable
**Purulent reaction scales**				
**Grade**	**Description**			
0	No exudate (except for blood); normal appearing wound bed			
1	Serous or slight turbid exudate; reddish wound clearly visualized			
2	Mild amount of whitish exudate; reddish wound bed visualized			
3	Moderate amount of whitish exudate; wound bed not visualized			
4	Moderate amount of yellowish exudate; exudate limited to wound bed			
5	Marked amount of gross pus; pus extending beyond wound edges			

In the first experiment, DR3 mice (*n* = 16) were challenged with 10^7^ CFU of *S. aureus* IDRL-7971, with 4 mice sacrificed on each of Days 2, 4, 6 and 8. Uninfected DR3 mice (*n* = 4) were sacrificed at Day 8. In the second experiment, DR3 mice were challenged with 10^7^ CFU of *S. aureus* RN6734/pRN7114 (SEB+, *n* = 11) or *S. aureus* RN6734/pRN7116 (SEB−, *n* = 9), with uninfected DR3 mice (*n* = 5) also studied; animals were sacrificed on Day 7. To examine the effect of the bacterial inoculum size on mortality, 10^8^ CFU of *S. aureus* RN6734/pRN7114 (*n* = 7) or *S. aureus* RN6734/pRN7116 (*n* = 3) were also tested on DR3 mice.

### 4.4. Flow Cytometry

Splenic mononuclear cells were stained with FITC-conjugated anti-Vβ6 (clone R4-7) or anti-Vβ8 (clone F23.1), phycoerythrin-conjugated CD4 (clone—GK1.5) or PerCP-conjugated CD8 (clone 53-6.7) (BD Pharmingen™, San Diego, CA, USA). Cells were analyzed by flow cytometry using FlowJo Software (Tree Star, Ashland, OR, USA).

### 4.5. Histopathology

Livers, lungs and kidneys were fixed in 10% formalin and embedded in paraffin. Thin sections stained with hematoxylin and eosin were evaluated in a blinded fashion by a pathologist (BSP). Sections from control slides were first examined to determine baseline (score of 0). Then, the rest of the slides were screened in a blinded fashion and given a score of either 1 or 2 based on the extent of inflammatory changes. For each group, 6 to 16 individual sections from different mice were examined and scored. An Olympus AX70 research microscope fitted with an Olympus DP70 camera (Olympus America, Center Valley, PA, USA) was used to acquire images.

### 4.6. Multiplex ELISA of Cytokines

Serum was collected at the time of sacrifice and stored at −80 °C. The levels of serum cytokines were determined using a multiplex suspension array system (Bio-plex^®^, Bio-Rad Laboratories, Hercules, CA, USA) following the manufacturer’s guidelines.

### 4.7. Statistical Analysis

Statistical analysis was performed using SPSS software (Version 21; SPSS, Chicago, IL, USA) and GraphPad Prism 5.0 (GraphPad Software, La Jolla, CA, USA). Exudate scores, morbidity scores, wound sizes, quantities of bacteria in skin and serum cytokine levels were compared by the Student *t*-test or the Mann-Whitney U-test. The exudate score over time was further analyzed using linear regression and compared using the F-test. *p*-values of <0.05 were considered statistically significant.
